# Pathological Features and Survival Outcomes of Young Patients with Operable Colon Cancer: Are They Homogeneous?

**DOI:** 10.1371/journal.pone.0102004

**Published:** 2014-07-08

**Authors:** Qingguo Li, Changhua Zhuo, Guoxiang Cai, Hongtu Zheng, Dawei Li, SanJun Cai

**Affiliations:** 1 Department of Colorectal Surgery, Fudan University Shanghai Cancer Center, Shanghai, People's Republic of China; 2 Department of Oncology, Shanghai Medical College, Fudan University, Shanghai, People's Republic of China; University of North Carolina School of Medicine, United States of America

## Abstract

**Objective:**

To compare the pathological features and survival outcomes at different age subgroups of young patients with colon cancer.

**Methods:**

Using Surveillance, Epidemiology, and End Results (SEER) population-based data, we identified 2,861 young patients with colon cancer diagnosed between 1988 and 2005 treated with surgery. Patients were divided into four groups: group 1 (below 25 years), group 2 (26–30 years), group 3 (31–35 years) and group 4 (36–40 years). Five-year cancer specific survival data were obtained. Kaplan-Meier methods were adopted and multivariable Cox regression models were built for the analysis of long-term survival outcomes and risk factors.

**Results:**

There were significant different among four groups in pathological grading, histological type, AJCC stage, current standard (≥12 lymph nodes retrieval), mean number of lymph nodes examined and positive lymph nodes (p<0.001). The 5-year cause specific survival was 71.0% in group 1, 75.1% in group 2, 80.6% in group 3 and 82.5% in group 4, which had significant difference in both univariate (P = 0.002) and multivariate analysis (P = 0.041).

**Conclusions:**

Young patients with colon cancer at age 18–40 years are essentially a heterogeneous group. Patients at age 31–35, 36–40 subgroups have more favorable clinicopathologic characteristics and better cancer specific survival than below 30 years.

## Introduction

Colorectal cancer (CRC) in young age is a topic issue in oncology for many reasons. First, the sharp increase in the number of young patients with CRC diagnosed in last decades reported in several countries. The 2010 Annual Report to the Nation on Cancer celebrated a steady decline in the incidence of CRC in USA [1]. In sharp contrast to overall trends, the incidence of CRC in young patients appears to be increasing [1], [2], [3].The incidence of the disease, considering patients aged between 20–40 years of age increased by 17% during the period between 1973 and 1999 [2]. Moreover, the prognosis of CRC in young patients remains much controversies. Majority of studies in the literature used the cutoff age of 40 years to denote a young patients with CRC [3], [4], [5], [6], [7]. Various studies have reported poorer prognosis among young patients with CRC [6], [8], [9]. Our previous study and some other authors demonstrated young patients with CRC treated with surgery appear to have a higher cancer specific survival (CSS) rate than elderly ones [7], [10], [11]. For young age is an inherent characteristic of wider age subgroups with potential heterogeneous.In this study, we updated our previous information about young patients with colon cancer (CC) and decided to evaluate four subgroups of patients according to four different age ranges, below 25 years, 26–30 years, 31–35 years and 36–40 years. Aim of our study was to analyze biological and clinical features and CSS of these four age-groups of young patients (below 40 years) with CC after surgery resection in Surveillance, Epidemiology, and End Results (SEER) population-based data.

## Patients and Methods

### Patients

The SEER Cancer Statistics Review (http://seer.cancer.gov/data/citation.html), a report on the most recent cancer incidence, mortality, survival, prevalence and lifetime risk statistics, is published annually by the Data Analysis and Interpretation Branch of the National Cancer Institute, MD, and USA. The current SEER database consists of 17 population-based cancer registries that represent approximately 28% of the population in the United States. The SEER data contain no identifiers and are publicly available for studies of cancer-based epidemiology and survival analysis [10], [12], [13], [14].

Cases of invasive CC (C18.0-19.9) diagnosed between 1988 and 2005 were extracted from the SEER database (SEER*Stat 8.1.2) according to the Site Recode classifications. Histological type were limited to adenocarcinoma (8150/3, 8210/3, 8261/3, 8263/3), mucinous adenocarcinoma (8480/3), and signet ring cell carcinoma (8490/3). Only patients aged between 18 and 40 years old and who's CC was a single primary tumor were included into the current study. Patients diagnosed after 2006 were excluded to ensure an adequate follow-up time. Other exclusion criterions were as follows: no lymph nodes (LNs) examined pathologically, synchronous distance metastases.

This study was based on the publicly available data from the SEER database and we had got the permission to access these research data (Reference number: 12768-Nov 2012). It didn't include interaction with human subjects or use personal identifying information. The study did not require informed consent and was approved by the Review Board of Fudan University Shanghai Cancer Center, Shanghai, China.

### Statistical analysis

Age, sex, race, extension of primary tumor invasion, total number of LNs examined, number of involved LNs, histological grade, survival time, and colon cancer-cause specific death (CCSS) were extracted from SEER database. All cases were restaged according to the criteria described in the American Joint Committee on Cancer (AJCC) Cancer Staging Manual (7th edition, 2010). And young patients with CC were divided into four subgroups according to four different age ranges: group 1 (below 25 years), group 2 (26–30 years), group 3 (31–35 years) and group 4 (36–40 years). The primary endpoint of this study was CCSS which was calculated from the date of diagnosis to the date of cancer specific death. Deaths attributed to the CC of interest are treated as events and deaths from other causes are treated as censored observation. Survival curves were generated using Kaplan-Meier estimates, differences between the curves were analyzed by log-rank test. Multivariable Cox regression models were built for analysis of risk factors for survival outcomes. All of the statistical analyses were done using the statistical software package SPSS for Windows, version 17 (SPSS Inc, Chicago, IL). Statistical significance was set at two-sided P<0.05.

## Results

### Patient characteristics

We identified 2,861 eligible young patients with CC in SEER database during the 18-year study period (between 1988 and 2005), which included 171 patients in group 1 (below 25 years), 375 patients in group 2 (26–30 years), 773 patients in group 3 (31–35 years) and 1542 patients in group 4 (36–40 years). There were 1,529 (53.4%) males and 1,332 (46.6%) females. The median age was 36. The median follow-up period was 87 (IQR 59-134) months. Patient demographics and pathological features are summarized in Table 1.

**Table 1:** Characteristics of Patients from SEER Database by age.

		Group 1	Group 2	Group 3	Group 4	
	Total	18–25	26–30	31–35	36–40	P value
Characteristic	(n = 2,861)	(n = 171)	(n = 375)	(n = 773)	(n = 1542)	
Media follow up (mo)	87	72	85	89	89	<0.001
(IQR)	59-134	32-103	44-130	60-135	61-138	
Years of diagnosis						0.154
1988–1993	432	14	62	114	242	
1994–1999	665	36	89	182	358	
2000–2003	1764	121	224	477	942	
Sex						0.284
male	1529	93	183	421	832	
female	1332	78	192	352	710	
Race						0.792
Caucasian	2047	117	264	566	1100	
African American	442	28	64	110	240	
Others*	365	26	47	94	198	
Pathological grading						0.002
High/Moderate	2042	114	243	541	1144	
Poor/undifferentiation	713	50	120	206	337	
Unknown	106	7	12	26	61	
Histological Type						<0.001
Adenocarcinoma	2267	115	281	630	1241	
Mucinous/Signet-ring cancer	587	55	91	142	299	
AJCC stage						0.002
I	251	12	19	67	153	
II	1184	60	141	324	659	
III	1426	99	215	382	730	
No. of LNs retrieval	19.34	24.21	20.32	19.45	18.50	<0.001
No. of LNs metastasis	2.17	3.07	2.62	2.27	1.90	<0.001
Current Standard						
<12	833	37	87	224	485	0.002
≥12	2028	134	288	549	1057	

*including other (American Indian/AK Native, Asian/Pacific Islander) and unknowns.

### Clinicopathological differences between the four groups

When compared among four subgroups, it was investigated that significant differences were found among the pathological grading (more poor or undifferentiation in grade in group 1, p = 0.002), histological type (more mucinous/signet-ring cancer in group 1, p<0.001), AJCC stage (more stage III in group 1, p = 0.002), current standard (more cases with ≥12 LNs dissected in group 1, p = 0.002). The mean number of LNs examined and positive LNs were 24.21 and 3.07 in group 1, 20.32 and 2.62 in group 2, 19.45 and 2.27 in group 3, 18.50 and 1.90 in group 4, respectively. All of them had statistical difference (p<0.001). As regard to year of diagnosis (p = 0.154), sex (p = 0.284) and race (p = 0.792), no significant differences between four groups were found. The difference in the median follow-up times, which is a reflection of the survival experience of these four groups, also had statistical difference (p<0.001). (Table 1)

### Impact of age on survival outcomes in young patients with CC

**Figure 1:**
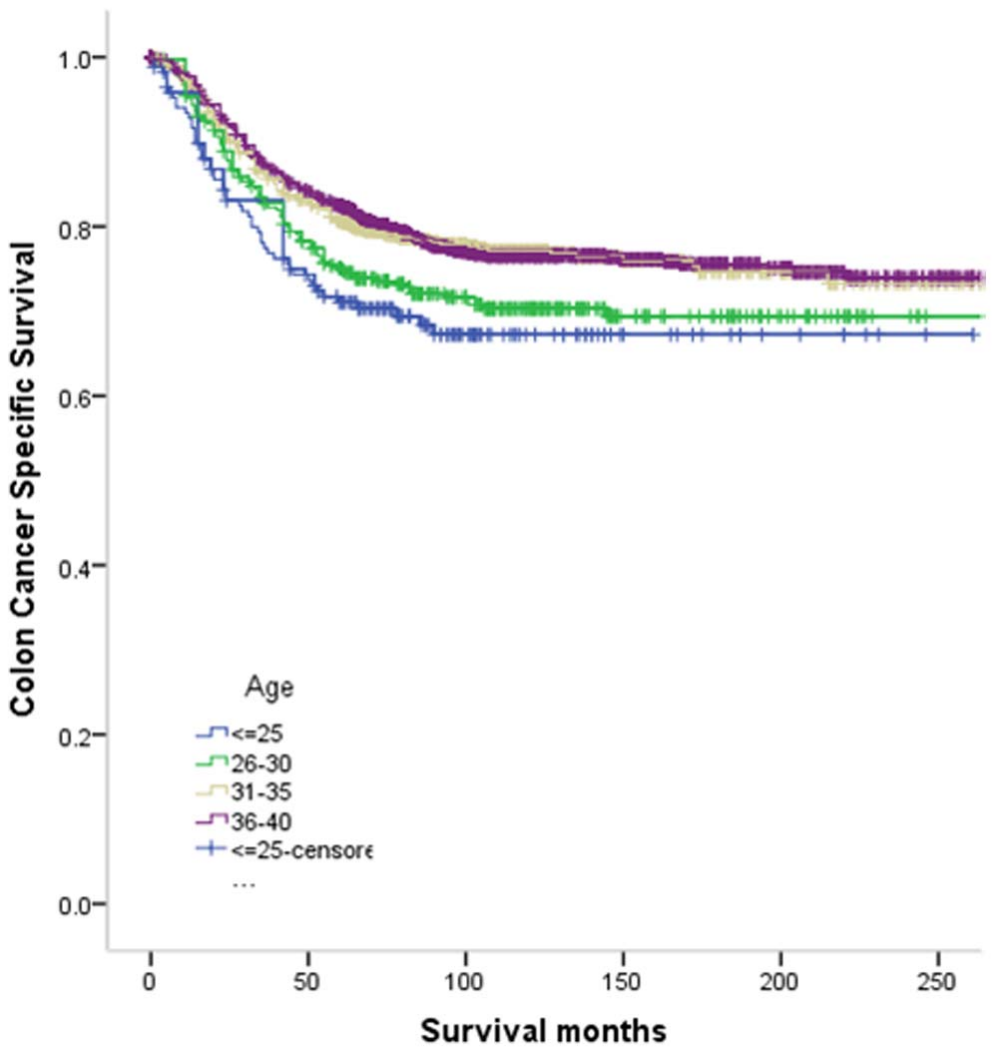
Survival curves in colon patients according to four age subgroups. Group 1 vs. group 2, *χ2 = 0.893, P = 0.345*; group 1 vs. group 3, *χ2 = 7.539, P = 0.006*. gropu 1 vs. group 4, *χ2 = 9.937, P = 0.002*; group 2 vs. group 3, *χ2 = 4.685, P = 0.030*. *group 3* vs. group 4, *χ2 = 7.052, P = 0.008*; group 3 vs. group 4, *χ2 = 0.075, P = 0.785*.

The 5-year CCSS was 71.0% in group 1, 75.1% in group 2, 80.6% in group 3 and 82.5% in group 4, which had significant difference in univariate log-rank test (P = 0.002) (Fig. 1). Besides, African race (P = 0.019), poor or undifferentiation tumor grade (P<0.001), mucinous/signet-ring cancer (P<0.001), higher AJCC stage (P<0.001), and less number in LNs dissection (p<0.001) were identified as significant risk factors for poor survival on univariate analysis (Table 2). When multivariate analysis with Cox regression was performed, we convinced the following five factors as independent prognostic factors (Table 3). These included age (group 3, HR 0.681, 95%CI 0.479–0.932, P = 0.017; group 4, HR 0.676, 95%CI 0.503–0.909, P = 0.010, using group 1 as reference), while the risk between group 1 and group 2 was not statistical difference (P = 0.186). Race (African American, HR 1.381,95%CI 1.130–1.687 P = 0.002; others, HR 1.14, 95%CI 0.885–1.403, P = 0.357, using Caucasian as reference), pathological grading (poor/undifferentiation tumor, HR 1.520, 95%CI 1.286–1.797, P<0.001, using high/moderate tumor as reference), AJCC stage (stage II, HR 5.076, 95%CI 2.241–11.496, P<0.001; stage III, HR 17.047, 95%CI 7.609–38.190, P<0.001, using stage I as reference), current standard (retrieval LNs≥12, HR 0.620, 95%CI 0.529–0.727, P<0.001, using retrieval LNs<12 as reference). And histological type of tumor was not a prognostic factor according to multivariate survival analyses (P = 0.057) (Tables 3).

**Table 2:** Univariate survival analyses of CC patients according to various clinicopathological variables.

Variable	n	5-year CCSS (%)	Log rank χ^2^ test	P
Years of diagnosis			5.398	0.067
1988–1993	432	76.8%		
1994–1999	665	79.5%		
2000–2003	1764	81.6%		
Sex			0.278	0.598
male	1529	80.8%		
female	1332	79.8%		
Age			15.261	0.002
≤25	171	71.0%		
26–30	375	75.1%		
31–35	773	80.6%		
36–40	1542	82.5%		
Race			7.933	0.019
Caucasian	2047	81.6%		
African American	442	76.4%		
Others*	365	77.4%		
Pathological grading			66.890	<0.001
High/Moderate	2042	84.4%		
Poor/undifferentiation	713	70.5%		
Unknown	106	66.9%		
Histological Type			18.858	<0.001
Adenocarcinoma	2267	82.0%		
Mucinous/Signet ring cancer	587	74.1%		
AJCC stage			289.312	<0.001
I	251	98.8%		
II	1184	90.3%		
III	1426	68.7%		
No. of LNs dissected			25.506	<0.001
<12	833	74.8%		
≥12	2028	82.6%		

*including other(American Indian/AK Native, Asian/Pacific Islander) and unknowns.

**Table 3:** Multivariate Cox model analyses of prognostic factors of CC.

Variable	Hazard Ratio	95%CI	P
Age			0.041
≤25	1.000	Reference	
26–30	0.797	0.569–1.116	0.186
31–35	0.681	0.497–0.932	0.017
36–40	0.676	0.503–0.909	0.010
Race			0.007
Caucasian	1.000	Reference	
African American	1.381	1.130–1.687	0.002
Others*	1.114	0.885–1.403	0.357
Pathological grading			<0.001
High/Moderate	1.000	Reference	
Poor/undifferentiation	1.520	1.286–1.797	<0.001
Unknown	2.137	1.526–2.993	<0.001
Histological Type			0.057
Adenocarcinoma	1.000	Reference	
Mucinous/Signet ring cancer	1.191	0.995–1.427	
AJCC stage			<0.001
I	1.000	Reference	
II	5.076	2.241–11.496	<0.001
III	17.047	7.609–38.190	<0.001
No. of LNs dissected			<0.001
<12	1.000	Reference	
≥12	0.620	0.529–0.727	

*including other (American Indian/AK Native, Asian/Pacific Islander) and unknowns.

## Discussion

Many studies evaluated biological behavior and risk of relapse and death in young patients with CC [3], [4], [5], [7], [8], [9], [10], [11]. Despite much research, CC in the young has not been well characterized, due to conflicting data in the literature. For example, various studies have reported poorer prognosis among young patients with CC [6], [8], [9], but our previous study and some recently published articles showed better CCSS in young patients after surgery than elderly ones [7], [10], [11]. These inconsistent could be explained by: First, the current definition of young CRC patients remains controversial. Although majority of studies in the literature used the cutoff age of 40 years to denote a young patients with CC [3], [4], [5], [6], [7], some other studies used the cutoff age of 30 years [4], [15], 25 years [16] or others [17], [18], [19]. Second, young age consisted wide age range of population, which maybe an inherent characteristic of heterogeneous, different composition of young subgroup may cause different results. Nonetheless no studies have evaluated both the clinicopathological features and CCSS of different strata of young patients with CC. In a smaller recent study, Schellerer et al evaluated the clinicopathologic characteristics and treatment outcomes of young patients (≤25 years) with CRC, and found sex and symptoms (abdominal pain and rectal bleeding) were significantly differed between child-adolescent (0 to 19 year) and young adult (20 to 25 year) and there also higher percentage of Dukes C/D stage and more proportion of moderated/poor differentiate cancer in young adult group [16], but authors didn't make statistical analysis of these and didn't consider the age-strata of patients between 26 and 40 years.

In this cohort, we found there were more patients with high/moderate grading, more adenocarcinoma and earlier stage (I/II) tumor, but smaller number of LNs retrieval and metastases with age increased in young patients, this difference had statistical significance. Patients with very young group (18–25 year) had similar 5 year CCSS with 26–30 year group, but significant lower than 31–35 and 36–40 year group, which confirmed our hypothesis that young patients was an inherently heterogeneous. In fact this did not exist in rectal cancer (Figure S1). Anders et al also revealed no significant differences in breast cancer in disease free survival among age groups younger than 30, 30–34, and 35–39 years [20].

Although this is a large population-based study, it has several potential limitations. First, the SEER database lacks several important tumor characteristics (eg, perineural invasion and lymphovascular invasion), cancer therapy (neoadjuvant and adjuvant, quality of surgery). Thus, our analyses could not adjust for these potential confounding factors. Second, this data include only patients who had undergone surgical resection for CC. As such, this group of patients can not represent CC patients who had irresectable tumors or refused surgical intervention for various reasons. Still, our study has its convincing power for its larger population based study.

In conclusion, our analysis of the SEER database revealed that the group of young patients with CC at age 18–40 years is essentially a heterogeneous group. Patients at age 31–35, 36–40 groups have more favorable clinicopathologic characteristics and better CCSS than below 30 years.

## Supporting Information

Figure S1 Survival curves in rectal cancer patients according to four age subgroups. Group 1 vs.group 2, *χ2 = 0.922, P = 0.337*; group 1 vs. group 3, *χ2 = 0.001, P = 0.973*. group 1 vs. group 4, *χ2 = 0.135, P = 0.714*; group 2 vs. group 3, *χ2 = 3.530, P = 0.060. group 3* vs. group 4, *χ2 = 1.535, P = 0.215*; group 3 vs. group 4, *χ2 = 1.105, P = 0.293*.
(TIF)
