# Supplementary figures and images for: Pathological Features and Survival Outcomes of Young Patients with Operable Colon Cancer: Are They Homogeneous?
Source: PLoS One. 2014 Jul 8;9(7):e102004. doi: 10.1371/journal.pone.0102004 (PMC4087023; doi:10.1371/journal.pone.0102004)

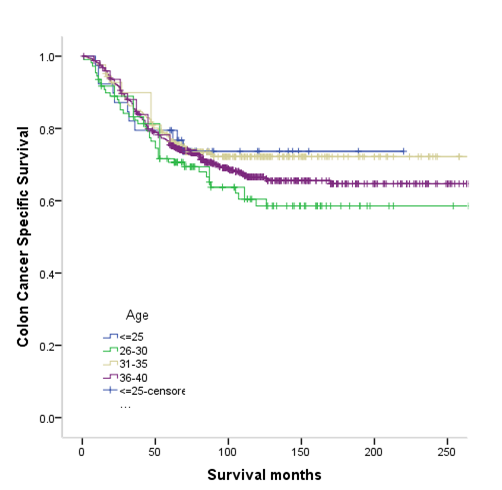

Supplement: Figure S1 — Survival curves in rectal cancer patients according to four age subgroups. Group 1 vs.group 2, χ2 = 0.922, P = 0.337; group 1 vs. group 3, χ2 = 0.001, P = 0.973. group 1 vs. group 4, χ2 = 0.135, P = 0.714; group 2 vs. group 3, χ2 = 3.530, P = 0.060. group 3 vs. group 4, χ2 = 1.535, P = 0.215; group 3 vs. group 4, χ2 = 1.105, P = 0.293. (TIF) [file pone.0102004.s001.tif]
